# Effectiveness of the national HIV pre-exposure prophylaxis (PrEP) programme among female sex workers in Rwanda: a retrospective cohort study

**DOI:** 10.1136/sextrans-2024-056189

**Published:** 2025-09-03

**Authors:** Eric Remera, Sabin Nsanzimana, Frédérique Chammartin, Heiner C Bucher

**Affiliations:** 1 Swiss Tropical and Public Health Institute, University of Basel Faculty of Medicine, Basel, BS, Switzerland; 2 Research Innovations and Data Science, Rwanda Biomedical Center, Kigali, Kigali City, Rwanda; 3 Republic of Rwanda Ministry of Health, Kigali, Rwanda; 4 Division of Clinical Epidemiology and Biostatistics, Department of Clinical Research, University Hospital Basel, Basel, Switzerland; 5 Division of Clinical Epidemiology, University Hospital Basel and University of Basel, Department of Clinical Research, University Hospital Basel, Basel, Switzerland

**Keywords:** Pre-Exposure Prophylaxis, HIV Infections, Sex Work

## Abstract

**Abstract:**

**Background:**

In 2018, Rwanda incorporated oral pre-exposure prophylaxis (PrEP) with tenofovir and emtricitabine (Truvada) into national HIV guidelines as part of a comprehensive HIV prevention programme for female sex workers (FSWs). This study assessed the impact of PrEP on HIV incidence among FSWs in urban Rwanda.

**Methods:**

We conducted a retrospective cohort study among HIV-negative FSWs aged≥18 years at 20 health facilities in Kigali from January 2019 to October 2021. All participants received standard HIV prevention services including routine condom distribution, peer education activities and the option to receive daily oral PrEP. Those who consented to receive PrEP formed the exposed group and those who declined or were not eligible to receive PrEP formed the control group. We used Cox regression to assess HIV seroconversion and logistic regression to assess retention in care.

**Results:**

Among 1897 FSWs (median age 30.1 years, IQR: 25.2–35.6), 1129 (59.5%) initiated PrEP. The HIV incidence rate was 0.40 per 100 person-years (PYs) among FSWs in the PrEP group versus 1.83 per 100 PYs for those in the non-PrEP group. In multivariate analysis, PrEP was associated with a reduced risk of HIV seroconversion (adjusted HR: 0.25; 95% CI: 0.09 to 0.71). Retention in the HIV prevention programme at 12 months was 77.6% among FSW who used PrEP versus 73.6% among non-users (adjusted OR: 1.29 (95% CI: 1.03 to 1.60).

**Conclusions:**

Oral PrEP was associated with reduced HIV risk of HIV seroconversion among FSWs in Kigali. However, the small number of HIV seroconversions and limitations of the observational design warrant cautious interpretation of study findings.

## Background

 Female sex workers (FSWs) are a key population in the global HIV epidemic due to their substantially elevated risk of infection, with HIV prevalence often reaching levels up to 20 times higher than those in the general population.^1^ Female sex work contributes significantly to new HIV infections in sub-Saharan Africa.^2^ In Rwanda, survey data from 2022 and 2023 estimated the median population FSW and sexually exploited minors at 37 647—representing 1.1% of females over age 15^3^—and found an HIV prevalence of 35.2%, 10 times higher than in the general population.^4^ A modelling study conducted in Sudan attributed up to 36% of new HIV infections in the general population to FSW and their clients.^5^

Pre-exposure prophylaxis (PrEP) is a highly effective tool for preventing HIV infection in key populations, providing significant protection when combined with other preventive strategies.^6^ Evidence suggests that PrEP can reduce the risk of HIV up to 80%.^7^ Real-world implementation of PrEP, however, faces significant obstacles. Adherence to PrEP among women in sub-Saharan Africa is particularly challenging due to structural barriers, stigma and complex socioeconomic factors. For instance, a study in Kigali found that 47% of FSW discontinued PrEP within the first 12 months of enrolment.^8^

Although the WHO has recommended PrEP as part of comprehensive HIV prevention programmes for individuals at high risk of HIV since 2015,^9^ large-scale PrEP implementation in sub-Saharan Africa has progressed slowly, in comparison to high-income countries. This lag is largely due to legal, social, political and economic factors impeding their implementation.^10^ In Rwanda, PrEP with tenofovir and emtricitabine (Truvada) was incorporated into HIV prevention guidelines in 2018.^11^

This study aims to evaluate the real-world effectiveness of PrEP in an urban setting within Rwanda’s existing healthcare system, providing crucial evidence for improving implementation strategies of PrEP targeting the key population of FSWs.

## Methods

### Study design

This retrospective cohort study included HIV-negative FSW receiving routine care at health centres in the Kigali and, who either accepted (exposed group) or declined (unexposed group) the offer of free-of-charge PrEP as part of an HIV prevention programme.

### Study setting

The study was conducted at 20 out of 59 health facilities in Kigali, where PrEP for FSWs was introduced in January 2019. Supported by the US President’s Emergency Plan for AIDS Relief, these facilities provide core HIV prevention services (regular HIV testing, sexual and reproductive health services and condom distribution) and specialised care approach for FSWs (peer education and psychosocial support). They also have staff trained to address the needs of key populations and provide services aimed at reducing stigma, preventing discrimination and improving health-seeking behaviour.

### Study participants

We included adult FSWs with confirmed HIV-negative status who received standard HIV prevention services between January 2019 and October 2021. These services included free condoms, monthly peer education on HIV prevention and HIV testing every 6 months. FSWs with a history of sexually transmitted infection (STI) symptoms in the past 12 months, inconsistent condom use, normal liver enzyme levels and an estimated glomerular filtration rate (eGFR)>60 mL/min/1.73 m² were invited to enrol in a daily, free of charge, PrEP uptake, consisting of tenofovir and emtricitabine (Truvada). Those who consented formed the PrEP-exposed group and received PrEP refills on a quarterly basis.

FSWs who declined PrEP, had no recent STI symptoms, reported consistent condom use or had reduced kidney function remained in the standard HIV prevention programme as the non-exposed group. PrEP users underwent HIV testing 1 month after initiation and every 3 months thereafter, while non-users received routine testing every 6 months or at follow-up completion. Participants who acquired HIV were immediately offered antiretroviral therapy per national guidelines (online supplemental figure S1).

### Data source

Data were recorded at health facilities in individual files containing demographic and clinical information at baseline and each follow-up visit. Health facility nurses entered the de-identified data into the District Health Information System-2, a free, open-source health data management platform managed by the national HIV programme. These data formed the foundation of this retrospective cohort study.

### Study variables and measurements

The primary outcome was HIV status at the end of follow-up. HIV testing followed Rwandan national guidelines, using two rapid point-of-care tests: the Alere HIV Combo assay for initial screening, confirmed by the HIV 1/2 STAT-PAK assay. Participants were followed until the first positive HIV test, death, loss to follow-up or 12 months—whichever occurred first.

The main predictor was PrEP exposure, alongside baseline socio-demographic and sexual/reproductive health variables from the HIV prevention programme. These included age (<25, 25–34, ≥35 years), education (none, primary, secondary/vocational, higher) and additional income beyond sex work (yes/no). Sexual health factors included the number of clients in the last 7 days (≤2, ≥3), HIV testing in the last year (yes/no), consistent condom use in the prior 3 months (yes/no) and history of syphilis during follow-up (yes/no). The secondary outcome was 12-month retention, defined as remaining in the programme without withdrawal or loss to follow-up.

To address potential bias arising from baseline differences between participants who initiated PrEP and those who did not, we applied inverse probability weighting (IPW). We ran balance checks that compared logistic regression models before and after applying IPW to evaluate its effectiveness (online supplemental table S1).

### Statistical methods

We report descriptive statistics for characteristics of FSWs who were exposed and unexposed to PrEP during the study period. Raw incidence rates are expressed in person-years (PYs) with 95% CIs. We constructed a multivariable Cox regression model using the specified variables to assess the effect of PrEP exposure on the hazard of HIV seroconversion. Variables with p value<0.05 in univariate analysis, as well as those deemed important based on expert opinion, were retained in a reduced multivariable Cox model to evaluate the effectiveness of PrEP among FSWs.

Cox regression models were weighted using IPW, and the proportional hazards assumption was evaluated through Schoenfeld residuals (online supplemental figure S2). The Global Schoenfeld test yielded a p value of 0.112, suggesting that the model satisfies the proportional hazards assumption (online supplemental table S2). We report adjusted HR (AHR) with 95% CIs. Retention in care was assessed by use of a logistic regression model, also weighted with IPW and using the same set of variables as the primary outcome. All analyses were conducted using R software, V.4.1.2.^12^

## Results

The study included 1897 HIV-negative FSWs with a median follow-up time of 12 months (IQR: 12–12) and a median age of 30.1 years (IQR: 25.2–35.6) (table 1). Of these, 1129 (59.5%) were eligible for the PrEP programme and consented to receive PrEP (exposed group) and 768/1897 (40.5%) did not consent to PrEP or were not eligible and were enrolled in the regular HIV prevention programme (non-exposed group).

**Table 1:** Participants’ characteristics, HIV incidence rates and reduced model for HIV seroconversion

	Participant’s characteristics (unweighted)			Retention	Incidence rate (IR)	Unadjusted model for HIV seroconversion	Reduced model for HIV seroconversion
	Total	Not exposed to PrEP	Exposed to PrEP				
	N	N (%)	N (%)	N (%)	N (IR per 100 PYs)	HR (95% CI)	AHR (95% CI)
**Overall (N**)	1897	768	1129	1441 (75.9)	20 (0.96)		
**Exposed to PrEP**							
Yes	1129 (59.5)			876 (77.6)	5 (0.40)	0.22 (0.08 to 0.63)	0.25 (0.09 to 0.71)
No	768 (40.5)			565 (73.6)	15 (1.83)	1.00	1.00
**Age (median (IQR))**	30.1 (25.2–35.6)	28.4 (24.2–34.4)	31.4 (26.2–36.4)				
Age group*							
<25 years	431 (23.0)	225 (29.5)	206 (18.5)	343 (79.6)	2 (0.42)	1.00	1.00
25–34 years	913 (48.6)	354 (46.5)	559 (50.1)	679 (74.4)	11 (1.00)	2.77 (0.61 to 12.6)	2.77 (0.60 to 12.8)
35 and above	533 (28.4)	183 (24)	350 (31.4)	408 (76.5)	7 (1.21)	3.64 (0.71 to 18.7)	2.87 (0.56 to 14.6)
**Other source of income besides sex work**							
Yes	448 (23.6)	159 (20.7)	289 (25.6)	350 (78.1)	6 (1.22)	1.00	
No	1449 (76.4)	609 (79.3)	840 (74.4)	1091 (75.3)	14 (0.89)	1.09 (0.37 to 3.21)	
Education level*							
None	211 (11.2)	73 (9.5)	138 (12.3)	160 (75.8)	2 (0.87)	1.00	1.00
Primary	1422 (75.2)	575 (75.1)	847 (75.3)	1079 (75.9)	16 (1.04)	0.99 (0.20 to 4.77)	0.93 (0.21 to 4.15)
Secondary and higher	258 (13.6)	118 (15.4)	140 (12.4)	196 (76)	2 (0.70)	0.81 (0.11 to 6.16)	0.80 (0.11 to 5.82)
**Number of partners in the last 7 days**							
≤2 partners	497 (26.2)	193 (25.1)	304 (26.9)	389 (78.3)	3 (0.55)	1.00	1.00
3 and more	1400 (73.8)	575 (74.9)	825 (73.1)	1052 (75.1)	17 (1.11)	1.88 (0.52 to 6.78)	1.61 (0.46 to 5.61)
**Previously tested for HIV**							
No	134 (7.1)	40 (5.2)	94 (8.3)	108 (80.6)	1 (0.67)	1.00	
Yes	1763 (92.9)	728 (94.8)	1035 (91.7)	1333 (75.6)	19 (0.99)	1.31 (0.17 to 9.95)	
**Consistent condom use in the last 3 months prior to enrolment**							
No	1339 (70.6)	579 (75.4)	760 (67.3)	1023 (76.4)	14 (0.96)	1.00	1.00
Yes	558 (29.4)	189 (24.6)	369 (32.7)	418 (74.9)	6 (0.98)	1.31 (0.47 to 3.70)	1.17 (0.41 to 3.35)
**History of syphilis during follow-up**							
No	1808 (95.3)	733 (95.4)	1075 (95.2)	1378 (76.2)	19 (0.96)	1.00	1.00
Yes	89 (4.7)	35 (4.6)	54 (4.8)	63 (70.8)	1 (1.05)	1.18 (0.16 to 8.91)	1.33 (0.18 to 10.0)

Variables that showed a significant association (p values<0.05) in the univariate model (exposed to PrEP) and those deemed important based on expert opinion (age, education level, number of sexual partners in last 7 days prior to the enrolment, history of syphilis during follow-up and consistent condom use in the 3 months prior to enrolment) were identified as key predictors. These were then included in the development of reduced multivariable Cox models to assess the effectiveness of PrEP among female sex workers.

* Variables (age group and educational level) with missing data.

AHR, adjusted HR; PrEP, pre-exposure prophylaxis; PYs, person-years.

Most FSW had attended only primary school (847/1129; 75.3% in the exposed group and 575/768; 75.1% in the non-exposed group)*,* reported no other source of income besides sex work (840/1129; 74.4% and 609/768; 79.3%, respectively) and had more than three sex partners in the last 7 days before the enrolment date (825/1129; 73.1% and 575/768; 74.9%). Prior HIV testing was reported by 1035/1129; 91.7% FSW in the exposed and 728/768; 94.8% in the non-exposed group.

During the follow-up period, 20 FSWs seroconverted, corresponding to an overall HIV incidence rate of 0.96 per 100 PYs (95% CI: 0.59 to 1.49). Incidence was higher among those not exposed to PrEP (1.83 per 100 PYs; 95% CI: 1.02 to 3.02) compared with those who were exposed to PrEP (0.40 per 100 PYs; 95% CI: 0.13 to 0.93) (table 1).

After adjusting for confounding factors, FSWs exposed to PrEP had a lower risk of HIV infection (adjusted HR: 0.25; 95% CI: 0.09 to 0.71) compared with those unexposed. This corresponds to a 12-month risk difference of –1.46% (95% CI: –3.05 to –0.50) between the two groups. Retention in the HIV prevention programme at 12 months was higher among FSWs exposed to PrEP (77.6%) compared with those unexposed (63.7%) (adjusted OR: 1.29; 95% CI: 1.03 to 1.60) (online supplemental table S3).

## Discussion

This study demonstrates the feasibility and underscores the urgency of scaling up PrEP alongside an existing HIV prevention programme for FSWs in an urban setting in Rwanda. FSWs who received PrEP had a lower HIV incidence rate (0.40 per 100 PYs) compared with those not on PrEP (1.83 per 100 PYs). However, the limited number of new infections and the potential biases inherent in the observational study design should be considered when interpreting these findings.

Older FSWs were more likely to acquire HIV, a trend that has also been observed in Tanzania.^13^ This may reflect cumulative risk exposure over time, fewer sources of income or reduced ability to negotiate safer sex practices, particularly among less educated and older FSWs.^14^ Participants with a history of syphilis had an increased risk of HIV infection, underscoring the need for integrating STI control into HIV prevention programmes. Interestingly, FSWs who self-reported consistent condom use in the 3 months prior to the study had a higher risk of HIV infection. This finding may be explained in part by behavioural changes linked to PrEP use, as some studies suggest that individuals on PrEP may perceive a reduced need for condoms, potentially increasing their vulnerability to HIV.^15^

This study has several limitations, including baseline differences between FSWs who enrolled in the PrEP programme and those who did not. Although multivariate analysis adjusted for some social and behavioural factors routinely measured in the HIV prevention programme, unmeasured confounders may have influenced the results. Differences in follow-up schedules for HIV testing could have introduced bias, potentially inflating HRs in favour of PrEP. Additionally, the enrolment process prioritised higher-risk FSWs for PrEP initiation, although this bias was addressed using IPW. Finally, some behavioural variables were collected only at baseline and may have changed during follow-up.

Despite these challenges, expanding PrEP access for high-risk FSWs, along with an emphasis on retention and adherence, remains essential. Prevention programmes should also target other high-risk populations and other geographical areas with a higher HIV burden, while continuing to promote condom use, education and economic empowerment to reduce risk.

Future research should investigate the factors contributing to high dropout rates and identify strategies to improve PrEP adherence. Long-acting PrEP formulations may offer a promising approach to enhance retention in prevention programmes.

## Supplementary material

10.1136/sextrans-2024-056189 online supplemental file 1
