# Supplementary material for: Effectiveness of the national HIV pre-exposure prophylaxis (PrEP) programme among female sex workers in Rwanda: a retrospective cohort study
Source: Sex Transm Infect. 2025 Sep 3;101(7):e056189. doi: 10.1136/sextrans-2024-056189 (PMC12573335; doi:10.1136/sextrans-2024-056189)
Supplement: online supplemental file 1 [file sextrans-101-7-s001.docx]

Supplementary Appendix

This appendix has been provided by the authors to give readers additional information about their work.

Supplement to: Effectiveness of the National HIV Pre-Exposure Prophylaxis (PrEP) program among female sex workers in Rwanda: A retrospective cohort study

Eric Remera^1,2,3^, Sabin Nsanzimana^4,^ Frédérique Chammartin^2^ and Heiner C. Bucher^2^

1. **Research Innovations and Data Science Division, Rwanda Biomedical Centre, Kigali, Rwanda**
2. **Division of Clinical Epidemiology and Biostatistics, Department of Clinical Research, University Hospital Basel and University of Basel, Basel, Switzerland**
3. **Swiss Tropical and Public Health Institute, Basel, Switzerland**
4. **Ministry of Health, Kigali, Rwanda**

Table of Contents

Contents

[***Supplementary Background:** 3](#_Toc192047532)

[Key Population ‘HIV prevention services’ 3](#_Toc192047533)

[***Supplementary Methods** 3](#_Toc192047534)

[Incidence rate calculation 3](#_Toc192047535)

[Censoring of observations 3](#_Toc192047536)

[Addressing Selection Bias in PrEP Uptake Among Female Sex Workers 3](#_Toc192047537)

[Participant recruitment flow 5](#_Toc192047538)

[***Supplementary results** 5](#_Toc192047539)

[Test for Proportional Hazard 5](#_Toc192047540)

[Retention in HIV prevention program 7](#_Toc192047541)

List of tables

[Table S1: Balance check for PrEP exposure 4](#_Toc192047543)

[Table S2: Proportional Hazard 6](#_Toc192047544)

[Table S3: Retention in HIV prevention program among FSWs, stratified by PrEP Exposure 8](#_Toc192047545)

List of figures

[Figure S1: Recruitment Flow diagram 5](#_Toc192047548)

[Figure S2: Schoenfeld residuals Plot 6](#_Toc192047549)

**Effectiveness of the National HIV Pre-Exposure Prophylaxis (PrEP) program among female sex workers in Rwanda: A retrospective cohort study**

***Supplementary Background:**

Key Population ‘HIV prevention services’

In Rwanda, a range of preventive services has been established to help female sex workers (FSWs) reduce their vulnerability to HIV and other sexually transmitted infections (STIs), while also promoting their overall health and well-being. These services aim to empower FSWs by providing tailored healthcare, education, and resources that enhance their safety and protect their rights. These include regular HIV testing, condom distribution, and HIV prevention education. Additionally, healthcare providers are specifically trained to address the unique needs of key populations and to deliver care in a way that reduces stigma and discrimination.

***Supplementary Methods**

Incidence rate calculation

The incidence rate of HIV seroconversion was calculated using a person-time approach, expressed as: **Incidence Rate**=Total *Number of New HIV Cases / Person-Time at Risk×100*

Person-time was measured in **person-years (PYs)** from the time of enrollment until HIV seroconversion, study end, loss to follow-up, or censoring.

Censoring of observations

Censoring occurred in the following cases:

- **Lost to follow-up (LTFU):** Participants who missed follow-up visits for more than six months were considered lost to follow-up.
- **Study completion without HIV seroconversion:** Participants who remained HIV-negative at the study's conclusion were right-censored at their last recorded follow-up.
- **Initiation of antiretroviral therapy (ART):** If an FSW became HIV-positive and initiated ART, follow-up time was censored at the date of diagnosis.

Addressing Selection Bias in PrEP Uptake Among Female Sex Workers

To address potential selection bias in our analysis, we recognized that female sex workers who initiated PrEP (exposed group) likely differed systematically at baseline from those who did not (unexposed group). These inherent differences could bias our estimates when evaluating the intervention's effect on outcomes.

We implemented Inverse Probability Weighting (IPW) to balance these groups on key baseline characteristics. This statistical approach creates a pseudo-population where the distribution of potential confounders is similar between exposed and unexposed participants, enabling more valid comparisons.

The accompanying table presents results from logistic regression models predicting PrEP uptake based on several covariates: *(age group, education, other source of income besides sex work, consistent condom use in the last three months prior the enrolment, number of partners in the last seven days, previously tested for HIV, history of syphilis during follow-up).*

We compare these models before and after applying IPW to demonstrate the effectiveness of our balancing approach. This weighting technique helps mitigate confounding and allows for more accurate estimation of the impact of PrEP in this population. **[Table S1]**

Table S1: Balance check for PrEP exposure

|  | **Unweighted model** | | **Weighted model** | |
| --- | --- | --- | --- | --- |
|  |  | |  | |
|  | **AoR** | **95% CI** | **AoR** | **95% CI** |
| **Age group** |  |  |  |  |
| <25 years | 1.00 |  | 1.00 |  |
| 25-34 years | 1.70 | [1.34- 2.17] | 1.10 | [0.75, 1.61] |
| 35 and above | 2.01 | [1.52- 2.68] | 1.07 | [0.68, 1.69] |
| **Education** |  |  |  |  |
| None | 1.00 |  | 1.00 |  |
| Primary | 0.85 | [0.62- 1.16] | 0.83 | [0.55, 1.26] |
| Secondary and higher | 0.74 | [0.50- 1.10] | 0.72 | [0.45, 1.16] |
| **Other source of income besides sex work** |  |  |  |  |
| yes | 1.00 |  | 1.00 |  |
| no | 0.81 | [0.64- 1.02] | 0.80 | [0.49, 1.31] |
| **# of partners in the last seven days** |  |  |  |  |
| <=2 partners | 1.00 |  | 1.00 |  |
| 3 and more | 0.96 | [0.77- 1.19] | 0.95 | [0.70, 1.30] |
| **Consistent condom use in the last three months prior to enrolment.** |  |  |  |  |
| No | 1.00 |  | 1.00 |  |
| Yes | 1.64 | [1.32- 2.04] | 1.63 | [1.10, 2.41] |
| **Previously tested for HIV** |  |  |  |  |
| No | 1.00 |  | 1.00 |  |
| Yes | 0.61 | [0.41- 0.91] | 0.59 | [0.31, 1.13] |
| **History of syphilis during follow up** |  |  |  |  |
| No | 1.00 |  | 1.00 |  |
| Yes | 1.09 | [0.69- 1.74] | 1.07 | [0.65, 1.76] |

AOR: Adjusted Odds Ratio

Participant recruitment flow

The study enrolled a total of 1,897 HIV-negative female sex workers (FSWs). Among them, 1,129 (59.5%) consented to receive PrEP and formed the exposed group, while 768 (40.5%) who did not consent to PrEP were enrolled in the standard HIV prevention program as the non-exposed group. Within the PrEP-exposed group, 253 FSWs were lost to follow-up, 871 tested HIV-negative, and 5 tested HIV-positive. In contrast, within the non-PrEP group, 203 FSWs were lost to follow-up, 550 tested HIV-negative, and 15 tested HIV-positive [Figure: S1]

Figure S1: Recruitment Flow diagram


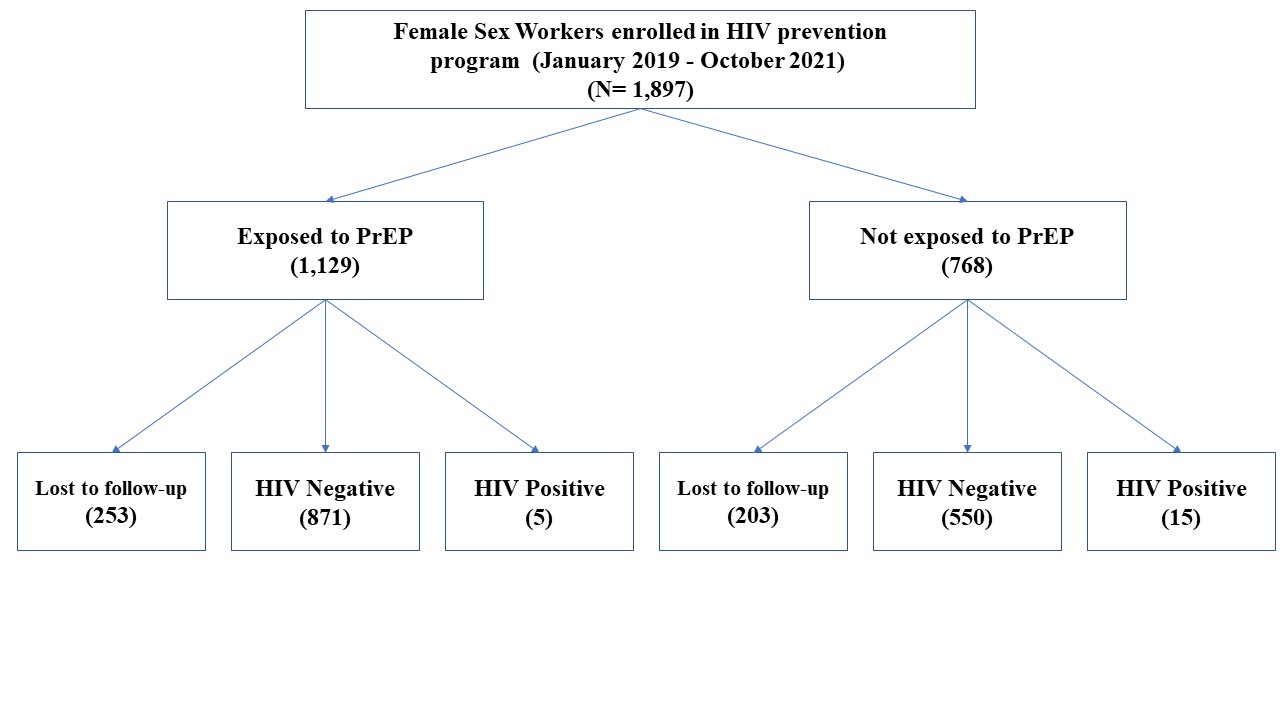


***Supplementary results**

Test for Proportional Hazard

The global test yielded a p-value of 0.112, indicating no significant violation of the proportional hazard’s assumption. Individual tests for each covariate (age group: p=0.072, number of partners in the last seven days: p=0.139, consistent condom use in the last three months prior to enrolment: p=0.549, education; p=0.207, and history of syphilis during follow up: p=0.589) also showed no significant violations. Visual inspection of the residual plots confirmed these findings, with residuals randomly distributed around zero across time for all variables. These results validate the appropriateness of the Cox proportional hazards model for our analysis.

Table S2: Proportional Hazard

|  | **Chi Square** | **Degree of freedom** | **P-value** |
| --- | --- | --- | --- |
| Exposed to PrEP | 1.582 | 1 | 0.208 |
| Age | 5.266 | 2 | 0.072 |
| Education | 3.154 | 2 | 0.207 |
| Number of partners in the last seven days | 2.194 | 1 | 0.139 |
| Consistent condom uses in the last three months prior to enrolment. | 0.36 | 1 | 0.549 |
| History of syphilis during follow-up | 0.292 | 1 | 0.589 |
| **GLOBAL** | **13.003** | **8** | **0.112** |

Figure S2: Schoenfeld residuals Plot


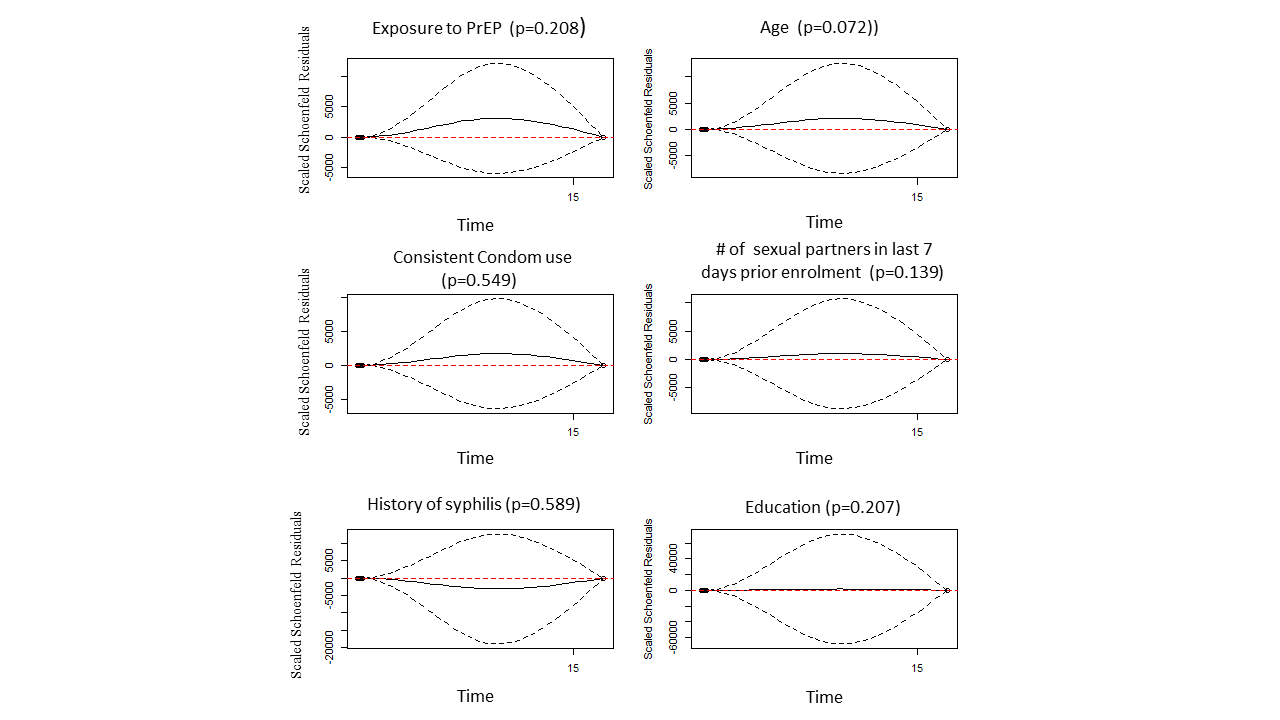


Retention in HIV prevention program

After 12 months of follow-up, **876 out of 1,129 (77.6%)** FSWs in the PrEP-exposed group and **565 out of 768 (73.6%)** in the non-exposed group remained enrolled in the HIV prevention program. FSWs who received PrEP were **more likely** to be retained in the program compared to those who were not exposed (**Adjusted odds ratio (AoR): 1.29, 95% CI: 1.03-1.60**). [Table S3].

Table S3: Retention in HIV prevention program among FSWs, stratified by PrEP Exposure

|  | **Retention in HIV Prevention program** | | | **Unadjusted Analysis** | | **Adjusted Analysis** | |
| --- | --- | --- | --- | --- | --- | --- | --- |
|  | **Overall** | **No Exposed to PrEP** | **Exposed to PrEP** | **Odds Ratio (OR)** | **95% Confidence Interval** | **Adjusted Odds Ratio (OR)** | **95% Confidence Interval** |
|  | **n (row %)** | **n (col %)** | **n (col %)** |  |  |  |  |
| **Overall (N)** | 1,441(75.9%) |  |  |  |  |  |  |
| **Exposed to PrEP** |  |  |  | 1.00 |  | 1.00 |  |
| Yes | 876 (77.6) |  |  | 1.24 | [1.01, 1.54] | 1.29 | [1.03, 1.60] |
| No | 565 (73.6) |  |  |  |  |  |  |
| **Age group** |  |  |  |  |  |  |  |
| <25 years | 343 (79.6) | 173.0 (76.9%) | 170.0 (82.5%) | 1.00 |  | 1.00 |  |
| 25-34 years | 679 (74.4) | 259.0 (73.2%) | 420.0 (75.1%) | 0.74 | [0.56, 0.98] | 0.72 | [0.54, 0.95] |
| 35 and above | 408 (76.5) | 130.0 (71.0%) | 278.0 (79.4%) | 0.84 | [0.61, 1.14] | 0.80 | [0.58, 1.09] |
| **Other source of income besides sex work** |  |  |  |  |  |  |  |
| yes | 350 (78.1) | 121.0 (76.1%) | 229.0 (79.2%) | 1.00 |  |  |  |
| no | 1,091 (75.3) | 444.0 (72.9%) | 647.0 (77.0%) | 0.85 | [0.66, 1.10] |  |  |
| **Education level** |  |  |  |  |  |  |  |
| None | 160 (75.8) | 53.0 (72.6%) | 107.0 (77.5%) |  |  |  |  |
| Primary | 1,079 (75.9) | 429.0 (74.6%) | 650.0 (76.7%) | 1.00 | [0.71, 1.40] |  |  |
| Secondary and higher | 196 (76) | 81.0 (68.6%) | 115.0 (82.1%) | 1.01 | [0.66, 1.54] |  |  |
| **Number of partners in the last seven days** |  |  |  |  |  |  |  |
| <=2 partners | 389 (78.3) | 150.0 (77.7%) | 239.0 (78.6%) | 1.00 |  |  |  |
| 3 and more | 1,052 (75.1) | 415.0 (72.2%) | 637.0 (77.2%) | 0.84 | [0.65, 1.07] |  |  |
| **Previously tested for HIV** |  |  |  |  |  |  |  |
| No | 108 (80.6) | 33 (82.5%) | 75 (79.8%) | 1.00 |  |  |  |
| Yes | 1,333 (75.6) | 532.0 (73.1%) | 801.0 (77.4%) | 0.75 | [0.47, 1.14] |  |  |
| **Consistent condom uses in the last three months prior to enrolment.** |  |  |  |  |  |  |  |
| No | 1,023 (76.4) | 421.0 (72.7%) | 602.0 (79.2%) | 1.00 |  |  |  |
| Yes | 418 (74.9) | 144.0 (76.2%) | 274.0 (74.3%) | 0.92 | [0.73, 1.16] |  |  |
| **History of syphilis during follow-up** |  |  |  |  |  |  |  |
| No | 1,378 (76.2) | 539.0 (73.5%) | 839.0 (78.0%) | 1.00 |  |  |  |
| Yes | 63 (70.8) | 26.0 (74.3%) | 37.0 (68.5%) | 0.76 | [0.48, 1.23] |  |  |
